# (2-Hy­droxy­eth­yl)triphenyl­phospho­nium chloride

**DOI:** 10.1107/S160053681100482X

**Published:** 2011-02-16

**Authors:** Ümit Ceylan, Hasan Tanak, Ercan Türkkan, Ömer Dereli, Orhan Büyükgüngör

**Affiliations:** a Department of Physics, Faculty of Arts & Science, Ondokuz Mayıs University, TR-55139 Kurupelit–Samsun, Turkey; b Department of Physics, Faculty of Arts & Science, Amasya University, TR-05100 Amasya, Turkey; c Department of Physics, A. K. Education Faculty, Selcuk University, TR-42090 Meram–Konya, Turkey

## Abstract

In the crystal structure of the title compound, C_20_H_20_OP^+^·Cl^−^, the cations and anions are linked by inter­molecular C—H⋯Cl and O—H⋯Cl hydrogen bonds into chains running parallel to the *b* axis. In the cation, the hy­droxy­ethyl group is disordered over two orientations with site-occupancy factors of 0.554 (4) and 0.446 (4).

## Related literature

For general background to the Wittig reaction, see: Wittig & Schöllkopf (1954 ▶); Wittig & Haag (1955 ▶). For the synthesis, applications and biological activity of triphenyl­phospho­nium compounds, see: Rideout *et al.* (1989 ▶); Cooper *et al.* (2001 ▶); Dubios & Lin (1978 ▶); Lou & Shang (2000 ▶); Calderon *et al.* (2008 ▶). For related structures, see: Shafiq *et al.* (2008 ▶); Wu *et al.* (2007 ▶).
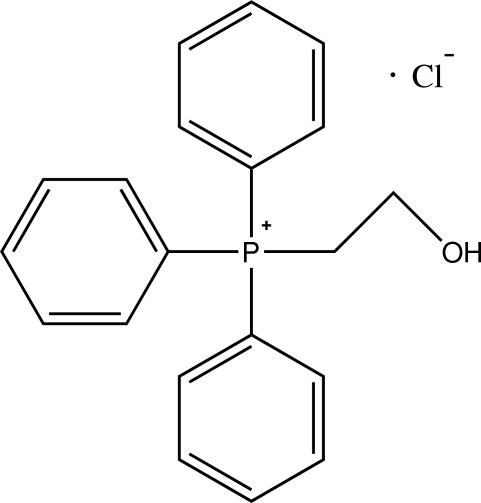


## Experimental

### 

#### Crystal data

C_20_H_20_OP^+^·Cl^−^
*M* *_r_* = 342.78
Monoclinic, 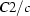

*a* = 14.1988 (4) Å
*b* = 12.5743 (3) Å
*c* = 19.7098 (6) Å
β = 92.510 (2)°
*V* = 3515.61 (17) Å^3^
*Z* = 8
Mo *K*α radiation
μ = 0.31 mm^−1^
*T* = 296 K
0.76 × 0.71 × 0.60 mm

#### Data collection

Stoe IPDS 2 diffractometer
Absorption correction: integration (*X-RED32*; Stoe & Cie, 2002 ▶) *T*_min_ = 0.599, *T*_max_ = 0.905
26668 measured reflections
3725 independent reflections
3317 reflections with *I* > 2σ(*I*)
*R*_int_ = 0.046

#### Refinement

*R*[*F* ^2^ > 2σ(*F* ^2^)] = 0.036
*wR*(*F* ^2^) = 0.101
*S* = 1.07
3725 reflections
230 parameters
35 restraints
H-atom parameters constrained
Δρ_max_ = 0.42 e Å^−3^
Δρ_min_ = −0.24 e Å^−3^

### 

Data collection: *X-AREA* (Stoe & Cie, 2002 ▶); cell refinement: *X-AREA*; data reduction: *X-RED32* (Stoe & Cie, 2002 ▶); program(s) used to solve structure: *SHELXS97* (Sheldrick, 2008 ▶); program(s) used to refine structure: *SHELXL97* (Sheldrick, 2008 ▶); molecular graphics: *ORTEP-3 for Windows* (Farrugia, 1997 ▶); software used to prepare material for publication: *WinGX* (Farrugia, 1999 ▶).

## Supplementary Material

Crystal structure: contains datablocks I, global. DOI: 10.1107/S160053681100482X/rz2551sup1.cif

Structure factors: contains datablocks I. DOI: 10.1107/S160053681100482X/rz2551Isup2.hkl

Additional supplementary materials:  crystallographic information; 3D view; checkCIF report

## Figures and Tables

**Table 1:** Hydrogen-bond geometry (Å, °)

*D*—H⋯*A*	*D*—H	H⋯*A*	*D*⋯*A*	*D*—H⋯*A*
C2—H2⋯Cl1	0.93	2.78	3.7009 (19)	171
C19—H19*C*⋯Cl1	0.97	2.73	3.6325 (18)	154
O1*B*—H1*B*⋯Cl1^i^	0.82	2.32	3.115 (4)	162
O1*A*—H1*A*⋯Cl1^i^	0.82	2.55	3.314 (4)	155
C19—H19*B*⋯Cl1^i^	0.97	2.78	3.5935 (19)	142

Symmetry code: (i) 
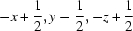
.

**Fig. 1.**
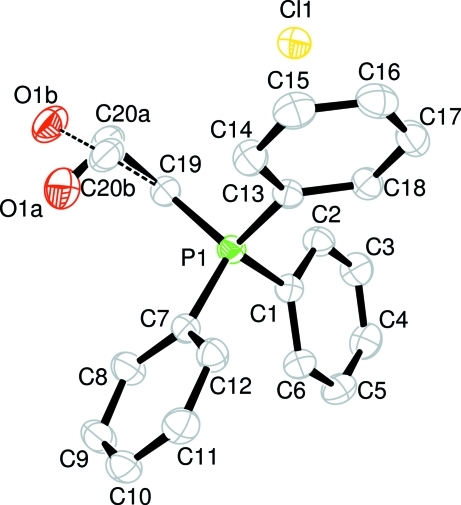
The structure of the title compound, with 30% probability displacement ellipsoids and the atom-numbering scheme. The H atoms are omitted for clarity.

**Fig. 2.**
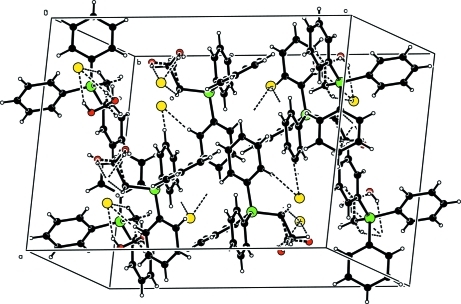
The crystal packing of the title compound. Intermolecular hydrogen bonds are drawn as dashed lines.

## supplementary crystallographic information

### Comment

Triphenylphosphonium compounds and their various derivatives are key reagents in the Wittig reactions and are used to convert aldehydes and ketones into alkenes (Wittig & Schöllkopf, 1954; Wittig & Haag, 1955), specifically in applications ranging from the synthesis of simple alkenes to the construction of complex biologically active molecules in the pharmaceutical research (Rideout *et al.*, 1989; Cooper *et al.*, 2001). They are also an important class of isoaromatic compounds and have widespread applications for their antimicrobial and anticancer activities (Dubios & Lin, 1978; Lou & Shang, 2000). In addition, phosphonium compounds enhance flame retardancy mainly in textile industry (Calderon *et al.*, 2008).

The title compound crystallizes with one cation and anion in the asymmetric unit (Fig. 1). In the molecule, the hydroxyethyl group (C19—C20—O1) is disordered over two orientations with site occupancy factors of 0.554 (4) and 0.446 (4), respectively. The dihedral angles between rings A (C1—C6), B (C7—C12) and C (C13—C18) are A/B = 73.79 (1)°, A/C = 67.88 (1)° and B/C = 70.96 (1)°. All the geometric parameters are in agreement with those observed in related compounds (Shafiq *et al.*, 2008; Wu *et al.*, 2007). The minimum separation between the P^+^ and Cl^-^ centres is 4.211 (1)Å. In the crystal structure, intermolecular C—H···Cl and C—H···O hydrogen bonds (Table 1) link the ions to form chains parallel to the *b* axis (Fig. 2).

### Experimental

(2-Hydroxyethyl)triphenylphosphonium chloride powder was purchased from Merck. Single crystals suitable for X-ray ananlysis were grown by slow evaporation of a concentrated acetonitrile solution.

### Refinement

H atoms were positioned geometrically and treated using a riding model, fixing the bond lengths at 0.93, 0.97 and 0.82 Å for aromatic CH, CH_2_, and OH groups, respectively. The displacement parameters of the H atoms were constrained as *U*_iso_(H) = 1.2*U*_eq_(C) or 1.5*U*_eq_(O). In the molecule, the hydroxyethyl group, (C19—C20—O1) is disordered over two orientations with site occupancy factors of 0.554 (4) and 0.446 (4). The disordered atoms were refined using the following restraints: SIMU, DELU and SADI (*SHELXL*; Sheldrick, 2008).

### Crystal data

**Table:**

C_20_H_20_OP^+^·Cl^−^	*F*(000) = 1440
*M**_r_* = 342.78	*D*_x_ = 1.295 Mg m^−^^3^
Monoclinic, *C*2/*c*	Mo *K*α radiation, λ = 0.71073 Å
Hall symbol: -C 2yc	Cell parameters from 36925 reflections
*a* = 14.1988 (4) Å	θ = 2.1–27.3°
*b* = 12.5743 (3) Å	µ = 0.31 mm^−^^1^
*c* = 19.7098 (6) Å	*T* = 296 K
β = 92.510 (2)°	Prism, colorless
*V* = 3515.61 (17) Å^3^	0.76 × 0.71 × 0.60 mm
*Z* = 8	

### Data collection

**Table:**

Stoe IPDS 2 diffractometer	3725 independent reflections
Radiation source: fine-focus sealed tube	3317 reflections with *I* > 2σ(*I*)
graphite	*R*_int_ = 0.046
Detector resolution: 6.67 pixels mm^-1^	θ_max_ = 26.8°, θ_min_ = 2.1°
rotation method scans	*h* = −17→17
Absorption correction: integration (*X-RED32*; Stoe & Cie, 2002)	*k* = −15→15
*T*_min_ = 0.599, *T*_max_ = 0.905	*l* = −24→24
26668 measured reflections	

### Refinement

**Table:**

Refinement on *F*^2^	Secondary atom site location: difference Fourier map
Least-squares matrix: full	Hydrogen site location: inferred from neighbouring sites
*R*[*F*^2^ > 2σ(*F*^2^)] = 0.036	H-atom parameters constrained
*wR*(*F*^2^) = 0.101	*w* = 1/[σ^2^(*F*_o_^2^) + (0.0495*P*)^2^ + 1.6584*P*] where *P* = (*F*_o_^2^ + 2*F*_c_^2^)/3
*S* = 1.07	(Δ/σ)_max_ = 0.001
3725 reflections	Δρ_max_ = 0.42 e Å^−^^3^
230 parameters	Δρ_min_ = −0.24 e Å^−^^3^
35 restraints	Extinction correction: *SHELXL97* (Sheldrick, 2008), Fc^*^=kFc[1+0.001xFc^2^λ^3^/sin(2θ)]^-1/4^
Primary atom site location: structure-invariant direct methods	Extinction coefficient: 0.0106 (6)

### Special details

**Table:**

Experimental. 360 frames, detector distance = 120 mm
Geometry. All e.s.d.'s (except the e.s.d. in the dihedral angle between two l.s. planes) are estimated using the full covariance matrix. The cell e.s.d.'s are taken into account individually in the estimation of e.s.d.'s in distances, angles and torsion angles; correlations between e.s.d.'s in cell parameters are only used when they are defined by crystal symmetry. An approximate (isotropic) treatment of cell e.s.d.'s is used for estimating e.s.d.'s involving l.s. planes.
Refinement. Refinement of *F*^2^ against ALL reflections. The weighted *R*-factor *wR* and goodness of fit *S* are based on *F*^2^, conventional *R*-factors *R* are based on *F*, with *F* set to zero for negative *F*^2^. The threshold expression of *F*^2^ > σ(*F*^2^) is used only for calculating *R*-factors(gt) *etc*. and is not relevant to the choice of reflections for refinement. *R*-factors based on *F*^2^ are statistically about twice as large as those based on *F*, and *R*- factors based on ALL data will be even larger.

### Fractional atomic coordinates and isotropic or equivalent isotropic displacement parameters (Å^2^)

**Table:**

	*x*	*y*	*z*	*U*_iso_*/*U*_eq_	Occ. (<1)
Cl1	0.25807 (3)	0.74387 (4)	0.18184 (2)	0.06015 (16)	
P1	0.22348 (3)	0.60936 (3)	0.37482 (2)	0.04301 (14)	
C1	0.34518 (11)	0.64165 (12)	0.39231 (8)	0.0449 (3)	
C2	0.39771 (12)	0.68183 (14)	0.33985 (9)	0.0545 (4)	
H2	0.3690	0.6949	0.2973	0.065*	
C3	0.49228 (13)	0.70205 (16)	0.35142 (11)	0.0633 (5)	
H3	0.5273	0.7292	0.3166	0.076*	
C4	0.53530 (13)	0.68242 (17)	0.41386 (11)	0.0655 (5)	
H4	0.5994	0.6958	0.4210	0.079*	
C5	0.48442 (14)	0.64332 (17)	0.46574 (10)	0.0652 (5)	
H5	0.5140	0.6305	0.5080	0.078*	
C6	0.38882 (12)	0.62269 (15)	0.45550 (9)	0.0543 (4)	
H6	0.3542	0.5963	0.4908	0.065*	
C7	0.18175 (11)	0.54012 (13)	0.44737 (8)	0.0457 (3)	
C8	0.21070 (14)	0.43627 (15)	0.46028 (9)	0.0606 (5)	
H8	0.2449	0.3994	0.4287	0.073*	
C9	0.18823 (16)	0.38821 (15)	0.52053 (10)	0.0665 (5)	
H9	0.2087	0.3193	0.5298	0.080*	
C10	0.13605 (13)	0.44101 (16)	0.56677 (9)	0.0608 (5)	
H10	0.1221	0.4082	0.6074	0.073*	
C11	0.10449 (13)	0.54206 (17)	0.55326 (9)	0.0617 (5)	
H11	0.0675	0.5769	0.5841	0.074*	
C12	0.12762 (12)	0.59252 (14)	0.49368 (9)	0.0527 (4)	
H12	0.1068	0.6615	0.4848	0.063*	
C13	0.15396 (11)	0.72717 (13)	0.36117 (8)	0.0450 (3)	
C14	0.05633 (12)	0.71654 (16)	0.34952 (10)	0.0581 (4)	
H14	0.0288	0.6494	0.3490	0.070*	
C15	0.00133 (13)	0.80521 (18)	0.33882 (10)	0.0667 (5)	
H15	−0.0634	0.7980	0.3308	0.080*	
C16	0.04150 (15)	0.90428 (17)	0.33992 (10)	0.0677 (5)	
H16	0.0037	0.9640	0.3329	0.081*	
C17	0.13779 (15)	0.91629 (15)	0.35143 (10)	0.0629 (5)	
H17	0.1646	0.9838	0.3522	0.076*	
C18	0.19394 (12)	0.82728 (13)	0.36184 (9)	0.0510 (4)	
H18	0.2587	0.8349	0.3693	0.061*	
C19	0.22152 (13)	0.52738 (14)	0.29984 (8)	0.0548 (4)	
H19A	0.2554	0.5656	0.2658	0.066*	0.554 (4)
H19B	0.2580	0.4641	0.3111	0.066*	0.554 (4)
H19C	0.2453	0.5679	0.2624	0.066*	0.446 (4)
H19D	0.2628	0.4668	0.3078	0.066*	0.446 (4)
O1A	0.0805 (2)	0.4345 (3)	0.31380 (16)	0.0879 (10)	0.554 (4)
H1A	0.1117	0.3827	0.3265	0.132*	0.554 (4)
C20A	0.1293 (4)	0.4906 (5)	0.2660 (4)	0.0667 (14)	0.554 (4)
H20A	0.0926	0.5514	0.2500	0.080*	0.554 (4)
H20B	0.1414	0.4454	0.2275	0.080*	0.554 (4)
O1B	0.1262 (3)	0.4096 (3)	0.23172 (16)	0.0766 (11)	0.446 (4)
H1B	0.1513	0.3564	0.2483	0.115*	0.446 (4)
C20B	0.1230 (4)	0.4883 (6)	0.2807 (6)	0.0672 (17)	0.446 (4)
H20C	0.0940	0.4603	0.3206	0.081*	0.446 (4)
H20D	0.0848	0.5471	0.2634	0.081*	0.446 (4)

### Atomic displacement parameters (Å^2^)

**Table:**

	*U*^11^	*U*^22^	*U*^33^	*U*^12^	*U*^13^	*U*^23^
Cl1	0.0621 (3)	0.0621 (3)	0.0561 (3)	0.0009 (2)	0.0013 (2)	0.00363 (19)
P1	0.0439 (2)	0.0434 (2)	0.0418 (2)	−0.00118 (16)	0.00250 (15)	0.00225 (15)
C1	0.0427 (8)	0.0448 (8)	0.0472 (8)	0.0022 (6)	0.0027 (6)	−0.0008 (6)
C2	0.0510 (9)	0.0598 (10)	0.0529 (9)	−0.0018 (8)	0.0037 (7)	0.0069 (7)
C3	0.0509 (10)	0.0718 (12)	0.0680 (11)	−0.0066 (9)	0.0106 (8)	0.0017 (9)
C4	0.0458 (9)	0.0720 (12)	0.0784 (13)	−0.0019 (8)	0.0006 (9)	−0.0108 (10)
C5	0.0562 (10)	0.0778 (13)	0.0604 (11)	0.0042 (9)	−0.0114 (8)	−0.0055 (9)
C6	0.0543 (9)	0.0613 (10)	0.0471 (8)	0.0014 (8)	0.0019 (7)	−0.0021 (7)
C7	0.0487 (8)	0.0458 (8)	0.0426 (7)	−0.0049 (6)	0.0026 (6)	0.0010 (6)
C8	0.0772 (12)	0.0519 (10)	0.0536 (9)	0.0067 (9)	0.0128 (9)	0.0036 (8)
C9	0.0862 (14)	0.0535 (10)	0.0602 (11)	0.0025 (9)	0.0076 (10)	0.0128 (8)
C10	0.0636 (11)	0.0698 (12)	0.0495 (9)	−0.0113 (9)	0.0074 (8)	0.0110 (8)
C11	0.0621 (10)	0.0727 (12)	0.0513 (9)	−0.0048 (9)	0.0144 (8)	−0.0046 (8)
C12	0.0568 (9)	0.0496 (9)	0.0522 (9)	−0.0016 (7)	0.0067 (7)	−0.0014 (7)
C13	0.0438 (8)	0.0482 (8)	0.0432 (7)	0.0017 (6)	0.0033 (6)	0.0035 (6)
C14	0.0468 (9)	0.0621 (10)	0.0654 (11)	−0.0026 (8)	0.0017 (8)	0.0010 (8)
C15	0.0475 (10)	0.0838 (14)	0.0683 (12)	0.0128 (9)	−0.0026 (8)	−0.0017 (10)
C16	0.0713 (12)	0.0689 (12)	0.0626 (11)	0.0249 (10)	0.0007 (9)	0.0045 (9)
C17	0.0767 (13)	0.0480 (9)	0.0644 (11)	0.0044 (9)	0.0073 (9)	0.0040 (8)
C18	0.0495 (9)	0.0510 (9)	0.0528 (9)	−0.0004 (7)	0.0043 (7)	0.0021 (7)
C19	0.0666 (10)	0.0499 (9)	0.0476 (8)	−0.0022 (8)	0.0013 (7)	−0.0016 (7)
O1A	0.0844 (19)	0.087 (2)	0.091 (2)	−0.0292 (16)	−0.0075 (15)	0.0052 (17)
C20A	0.080 (2)	0.0603 (19)	0.058 (3)	−0.0002 (17)	−0.0162 (18)	−0.0101 (17)
O1B	0.086 (2)	0.075 (2)	0.067 (2)	−0.0013 (17)	−0.0132 (16)	−0.0231 (16)
C20B	0.073 (2)	0.064 (2)	0.063 (4)	0.003 (2)	−0.018 (2)	−0.014 (2)

### Geometric parameters (Å, °)

**Table:**

P1—C1	1.7938 (16)	C13—C18	1.381 (2)
P1—C13	1.7939 (16)	C13—C14	1.401 (2)
P1—C7	1.7968 (15)	C14—C15	1.372 (3)
P1—C19	1.8009 (17)	C14—H14	0.9300
C1—C6	1.387 (2)	C15—C16	1.370 (3)
C1—C2	1.396 (2)	C15—H15	0.9300
C2—C3	1.376 (2)	C16—C17	1.384 (3)
C2—H2	0.9300	C16—H16	0.9300
C3—C4	1.372 (3)	C17—C18	1.384 (2)
C3—H3	0.9300	C17—H17	0.9300
C4—C5	1.369 (3)	C18—H18	0.9300
C4—H4	0.9300	C19—C20A	1.515 (4)
C5—C6	1.388 (3)	C19—C20B	1.516 (4)
C5—H5	0.9300	C19—H19A	0.9700
C6—H6	0.9300	C19—H19B	0.9700
C7—C12	1.385 (2)	C19—H19C	0.9700
C7—C8	1.389 (2)	C19—H19D	0.9700
C8—C9	1.382 (2)	O1A—C20A	1.386 (8)
C8—H8	0.9300	O1A—H1A	0.8200
C9—C10	1.371 (3)	C20A—H20A	0.9700
C9—H9	0.9300	C20A—H20B	0.9700
C10—C11	1.370 (3)	O1B—C20B	1.383 (8)
C10—H10	0.9300	O1B—H1B	0.8200
C11—C12	1.387 (2)	C20B—H20C	0.9700
C11—H11	0.9300	C20B—H20D	0.9700
C12—H12	0.9300		
			
C1—P1—C13	111.16 (7)	C15—C14—H14	120.0
C1—P1—C7	107.77 (7)	C13—C14—H14	120.0
C13—P1—C7	108.73 (7)	C16—C15—C14	120.17 (18)
C1—P1—C19	105.51 (8)	C16—C15—H15	119.9
C13—P1—C19	111.16 (8)	C14—C15—H15	119.9
C7—P1—C19	112.45 (8)	C15—C16—C17	120.62 (18)
C6—C1—C2	119.68 (15)	C15—C16—H16	119.7
C6—C1—P1	121.49 (12)	C17—C16—H16	119.7
C2—C1—P1	118.76 (12)	C18—C17—C16	119.63 (18)
C3—C2—C1	119.56 (17)	C18—C17—H17	120.2
C3—C2—H2	120.2	C16—C17—H17	120.2
C1—C2—H2	120.2	C13—C18—C17	120.12 (16)
C4—C3—C2	120.56 (18)	C13—C18—H18	119.9
C4—C3—H3	119.7	C17—C18—H18	119.9
C2—C3—H3	119.7	C20A—C19—P1	121.2 (4)
C5—C4—C3	120.38 (18)	C20B—C19—P1	111.8 (4)
C5—C4—H4	119.8	C20A—C19—H19A	107.0
C3—C4—H4	119.8	C20B—C19—H19A	118.0
C4—C5—C6	120.22 (18)	P1—C19—H19A	107.0
C4—C5—H5	119.9	C20A—C19—H19B	107.0
C6—C5—H5	119.9	C20B—C19—H19B	105.6
C1—C6—C5	119.60 (17)	P1—C19—H19B	107.0
C1—C6—H6	120.2	H19A—C19—H19B	106.8
C5—C6—H6	120.2	C20A—C19—H19C	98.6
C12—C7—C8	119.65 (15)	C20B—C19—H19C	109.3
C12—C7—P1	120.38 (13)	P1—C19—H19C	109.3
C8—C7—P1	119.74 (12)	H19B—C19—H19C	113.9
C9—C8—C7	119.39 (17)	C20A—C19—H19D	109.6
C9—C8—H8	120.3	C20B—C19—H19D	109.3
C7—C8—H8	120.3	P1—C19—H19D	109.3
C10—C9—C8	120.75 (18)	H19A—C19—H19D	100.8
C10—C9—H9	119.6	H19C—C19—H19D	107.9
C8—C9—H9	119.6	O1A—C20A—C19	107.7 (5)
C11—C10—C9	120.12 (16)	O1A—C20A—H20A	110.2
C11—C10—H10	119.9	C19—C20A—H20A	110.2
C9—C10—H10	119.9	O1A—C20A—H20B	110.2
C10—C11—C12	120.10 (17)	C19—C20A—H20B	110.2
C10—C11—H11	120.0	H20A—C20A—H20B	108.5
C12—C11—H11	120.0	C20B—O1B—H1B	109.5
C7—C12—C11	119.94 (17)	O1B—C20B—C19	110.3 (6)
C7—C12—H12	120.0	O1B—C20B—H20C	109.6
C11—C12—H12	120.0	C19—C20B—H20C	109.6
C18—C13—C14	119.46 (16)	O1B—C20B—H20D	109.6
C18—C13—P1	121.88 (12)	C19—C20B—H20D	109.6
C14—C13—P1	118.66 (13)	H20C—C20B—H20D	108.1
C15—C14—C13	120.00 (18)		
			
C13—P1—C1—C6	112.88 (14)	P1—C7—C12—C11	−172.90 (14)
C7—P1—C1—C6	−6.17 (16)	C10—C11—C12—C7	0.8 (3)
C19—P1—C1—C6	−126.51 (14)	C1—P1—C13—C18	2.53 (16)
C13—P1—C1—C2	−70.32 (15)	C7—P1—C13—C18	121.01 (14)
C7—P1—C1—C2	170.63 (13)	C19—P1—C13—C18	−114.68 (15)
C19—P1—C1—C2	50.29 (15)	C1—P1—C13—C14	−177.47 (13)
C6—C1—C2—C3	0.1 (3)	C7—P1—C13—C14	−58.99 (15)
P1—C1—C2—C3	−176.77 (14)	C19—P1—C13—C14	65.32 (15)
C1—C2—C3—C4	0.4 (3)	C18—C13—C14—C15	0.1 (3)
C2—C3—C4—C5	−0.6 (3)	P1—C13—C14—C15	−179.91 (15)
C3—C4—C5—C6	0.3 (3)	C13—C14—C15—C16	−0.4 (3)
C2—C1—C6—C5	−0.4 (3)	C14—C15—C16—C17	0.3 (3)
P1—C1—C6—C5	176.39 (14)	C15—C16—C17—C18	0.1 (3)
C4—C5—C6—C1	0.2 (3)	C14—C13—C18—C17	0.3 (2)
C1—P1—C7—C12	101.87 (14)	P1—C13—C18—C17	−179.67 (13)
C13—P1—C7—C12	−18.72 (16)	C16—C17—C18—C13	−0.4 (3)
C19—P1—C7—C12	−142.27 (14)	C1—P1—C19—C20A	−175.4 (3)
C1—P1—C7—C8	−72.50 (16)	C13—P1—C19—C20A	−54.8 (3)
C13—P1—C7—C8	166.91 (14)	C7—P1—C19—C20A	67.4 (3)
C19—P1—C7—C8	43.36 (17)	C1—P1—C19—C20B	176.9 (4)
C12—C7—C8—C9	−2.6 (3)	C13—P1—C19—C20B	−62.5 (4)
P1—C7—C8—C9	171.81 (16)	C7—P1—C19—C20B	59.6 (4)
C7—C8—C9—C10	1.5 (3)	C20B—C19—C20A—O1A	−20 (2)
C8—C9—C10—C11	0.8 (3)	P1—C19—C20A—O1A	−57.9 (5)
C9—C10—C11—C12	−2.0 (3)	C20A—C19—C20B—O1B	47 (2)
C8—C7—C12—C11	1.5 (3)	P1—C19—C20B—O1B	−168.2 (5)

### Hydrogen-bond geometry (Å, °)

**Table:**

*D*—H···*A*	*D*—H	H···*A*	*D*···*A*	*D*—H···*A*
C2—H2···Cl1	0.93	2.78	3.7009 (19)	171
C19—H19C···Cl1	0.97	2.73	3.6325 (18)	154
O1B—H1B···Cl1^i^	0.82	2.32	3.115 (4)	162
O1A—H1A···Cl1^i^	0.82	2.55	3.314 (4)	155
C19—H19B···Cl1^i^	0.97	2.78	3.5935 (19)	142

Symmetry codes: (i) −*x*+1/2, *y*−1/2, −*z*+1/2.
